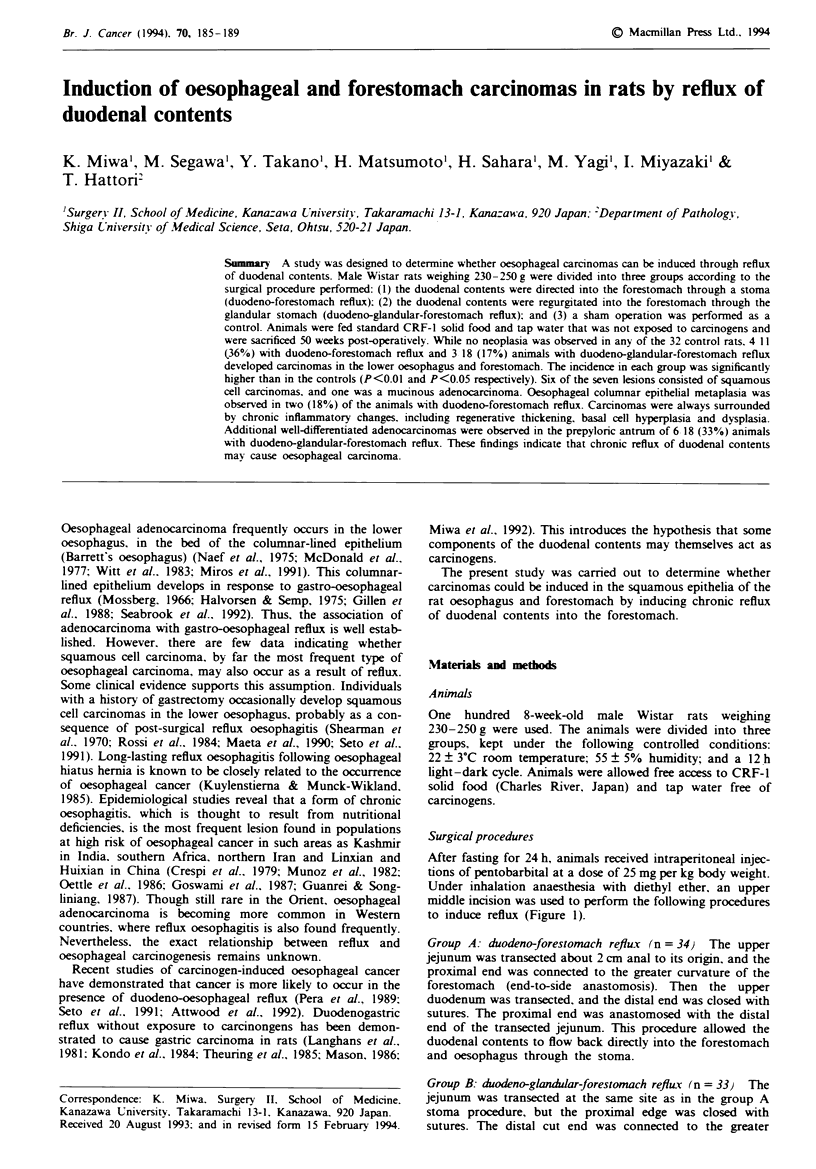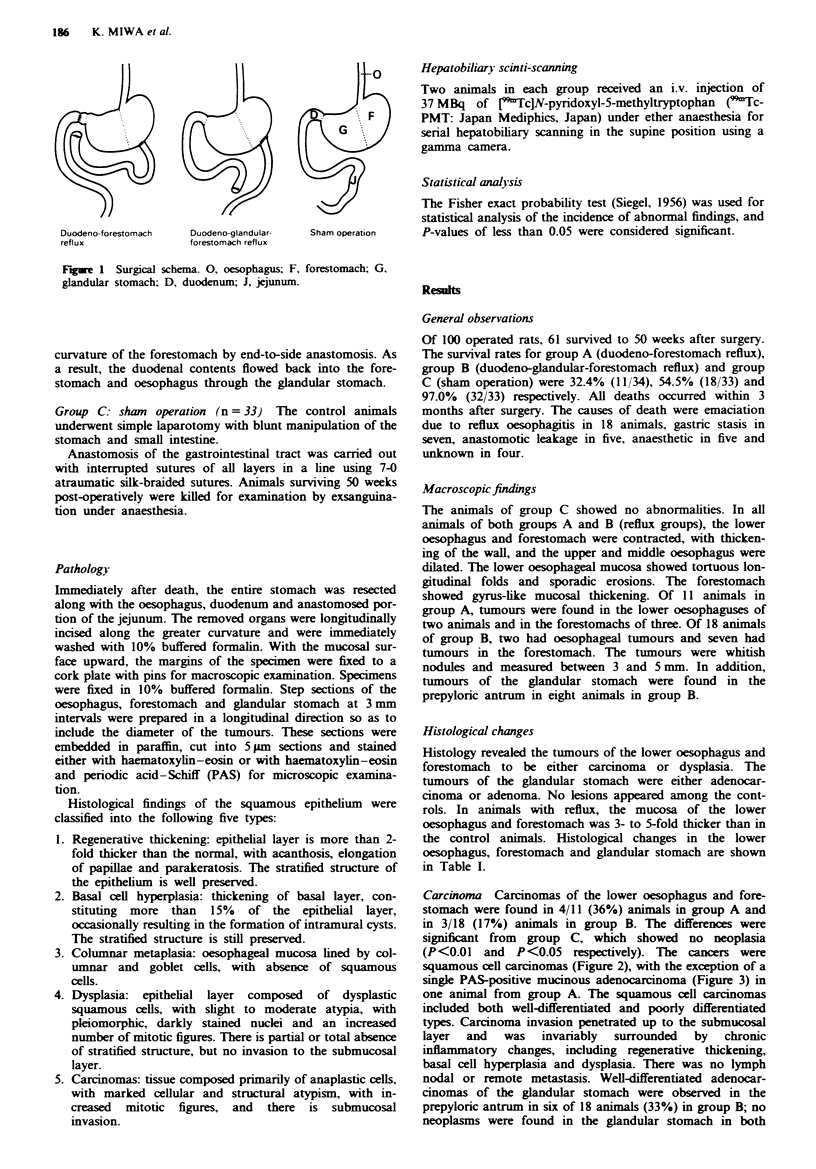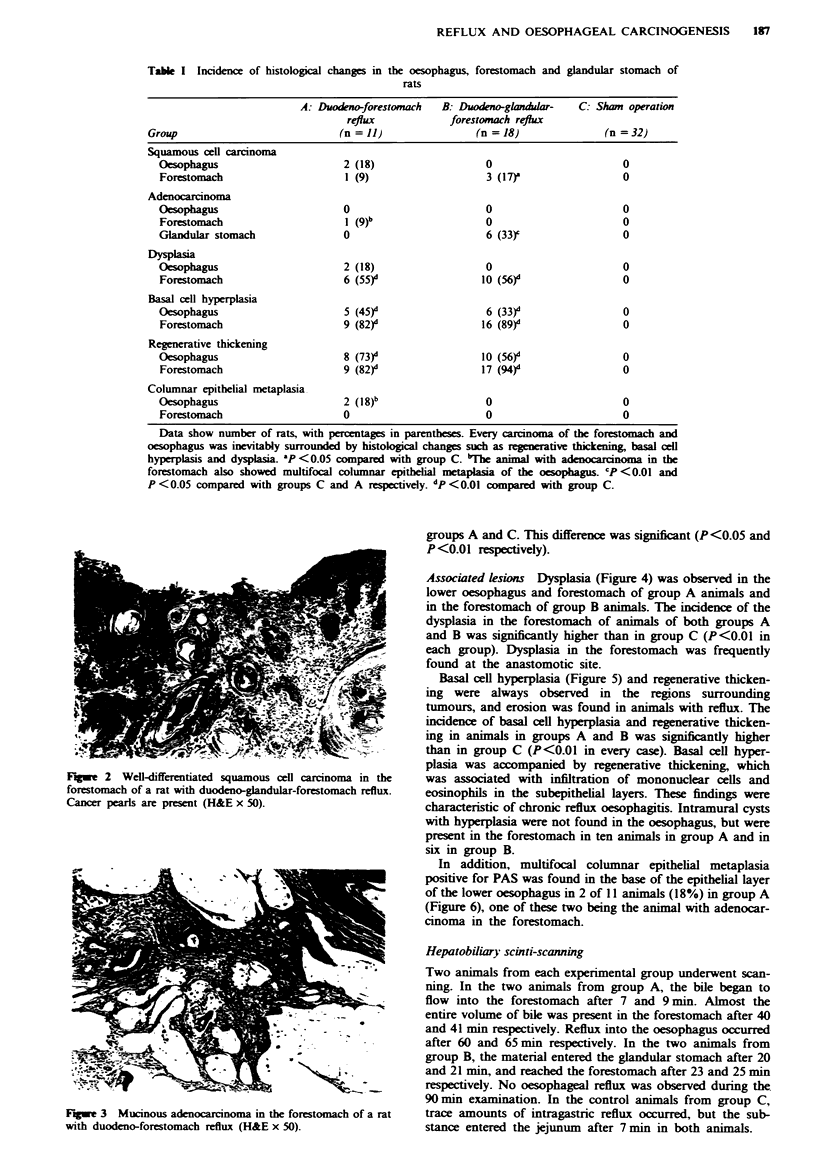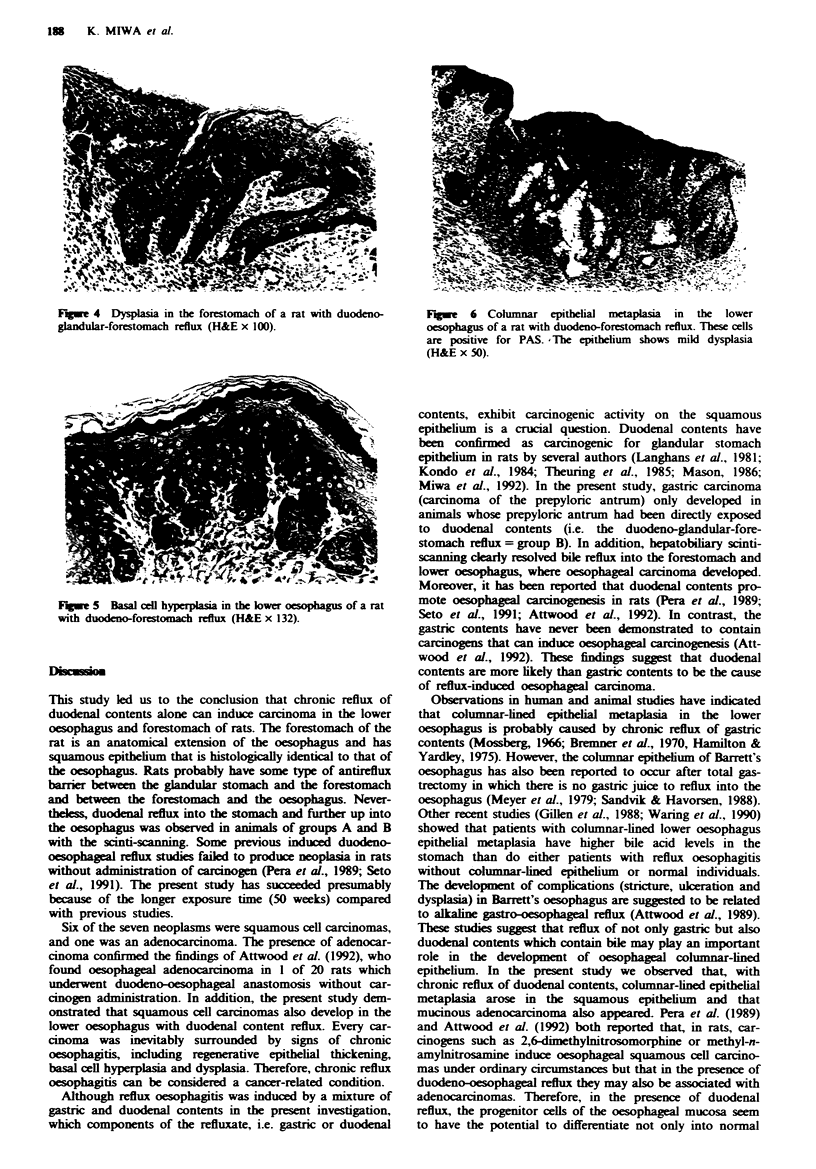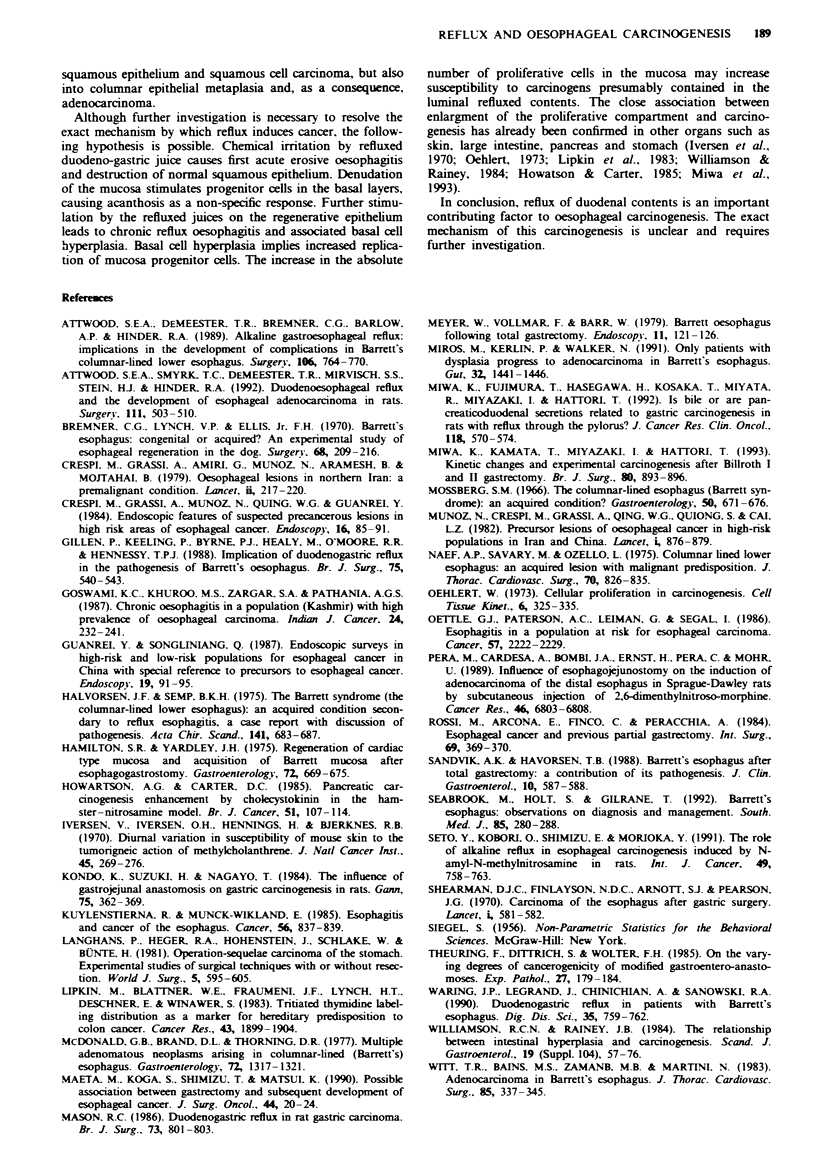# Induction of oesophageal and forestomach carcinomas in rats by reflux of duodenal contents.

**DOI:** 10.1038/bjc.1994.277

**Published:** 1994-08

**Authors:** K. Miwa, M. Segawa, Y. Takano, H. Matsumoto, H. Sahara, M. Yagi, I. Miyazaki, T. Hattori

**Affiliations:** School of Medicine, Kanazawa University, Japan.

## Abstract

**Images:**


Br. J. Cancer (1994), 70, 185-189 © Macmillan Press Ltd., 1994

### Induction of oesophageal and forestomach carcinomas in rats by reflux of duodenal contents

K. Miwa[1], M. Segawa[1], Y. Takano[1], H. Matsumoto[1], H. Sahara[1], M. Yagi[1], I. Miyazaki[1] & T. Hattori[2]

[1]*Surgery II, School of Medicine, Kanazawa University, Takaramachi 13-1, Kanazawa, 920 Japan;* [2]*Department of Pathology, Shiga University of Medical Science, Seta, Ohtsu, 520-21 Japan.*

**Summary** A study was designed to determine whether oesophageal carcinomas can be induced through reflux of duodenal contents. Male Wistar rats weighing 230-250 g were divided into three groups according to the surgical procedure performed: (1) the duodenal contents were directed into the forestomach through a stoma (duodeno-forestomach reflux); (2) the duodenal contents were regurgitated into the forestomach through the glandular stomach (duodeno-glandular-forestomach reflux); and (3) a sham operation was performed as a control. Animals were fed standard CRF-1 solid food and tap water that was not exposed to carcinogens and were sacrificed 50 weeks post-operatively. While no neoplasia was observed in any of the 32 control rats, 4/11 (36%) with duodeno-forestomach reflux and 3/18 (17%) animals with duodeno-glandular-forestomach reflux developed carcinomas in the lower oesophagus and forestomach. The incidence in each group was significantly higher than in the controls ($P<0.01$ and $P<0.05$ respectively). Six of the seven lesions consisted of squamous cell carcinomas, and one was a mucinous adenocarcinoma. Oesophageal columnar epithelial metaplasia was observed in two (18%) of the animals with duodeno-forestomach reflux. Carcinomas were always surrounded by chronic inflammatory changes, including regenerative thickening, basal cell hyperplasia and dysplasia. Additional well-differentiated adenocarcinomas were observed in the prepyloric antrum of 6/18 (33%) animals with duodeno-glandular-forestomach reflux. These findings indicate that chronic reflux of duodenal contents may cause oesophageal carcinoma.

Oesophageal adenocarcinoma frequently occurs in the lower oesophagus, in the bed of the columnar-lined epithelium (Barrett's oesophagus) (Naef *et al*., 1975; McDonald *et al*., 1977; Witt *et al*., 1983; Miros *et al*., 1991). This columnar-lined epithelium develops in response to gastro-oesophageal reflux (Mossberg, 1966; Halvorsen & Semp, 1975; Gillen *et al*., 1988; Seabrook *et al*., 1992). Thus, the association of adenocarcinoma with gastro-oesophageal reflux is well established. However, there are few data indicating whether squamous cell carcinoma, by far the most frequent type of oesophageal carcinoma, may also occur as a result of reflux. Some clinical evidence supports this assumption. Individuals with a history of gastrectomy occasionally develop squamous cell carcinomas in the lower oesophagus, probably as a consequence of post-surgical reflux oesophagitis (Shearman *et al*., 1970; Rossi *et al*., 1984; Maeta *et al*., 1990; Seto *et al*., 1991). Long-lasting reflux oesophagitis following oesophageal hiatus hernia is known to be closely related to the occurrence of oesophageal cancer (Kuylenstierna & Munck-Wikland, 1985). Epidemiological studies reveal that a form of chronic oesophagitis, which is thought to result from nutritional deficiencies, is the most frequent lesion found in populations at high risk of oesophageal cancer in such areas as Kashmir in India, southern Africa, northern Iran and Linxian and Huixian in China (Crespi *et al*., 1979; Munoz *et al*., 1982; Oettle *et al*., 1986; Goswami *et al*., 1987; Guanrei & Songliniang, 1987). Though still rare in the Orient, oesophageal adenocarcinoma is becoming more common in Western countries, where reflux oesophagitis is also found frequently. Nevertheless, the exact relationship between reflux and oesophageal carcinogenesis remains unknown.

Recent studies of carcinogen-induced oesophageal cancer have demonstrated that cancer is more likely to occur in the presence of duodeno-oesophageal reflux (Pera *et al*., 1989; Seto *et al*., 1991; Attwood *et al*., 1992). Duodenogastric reflux without exposure to carcinongens has been demonstrated to cause gastric carcinoma in rats (Langhans *et al*., 1981; Kondo *et al*., 1984; Theuring *et al*., 1985; Mason, 1986; Miwa *et al*., 1992). This introduces the hypothesis that some components of the duodenal contents may themselves act as carcinogens.

The present study was carried out to determine whether carcinomas could be induced in the squamous epithelia of the rat oesophagus and forestomach by inducing chronic reflux of duodenal contents into the forestomach.

### Materials and methods

### *Animals*

One hundred 8-week-old male Wistar rats weighing 230-250 g were used. The animals were divided into three groups, kept under the following controlled conditions: 22 ± 3°C room temperature; 55 ± 5% humidity; and a 12 h light-dark cycle. Animals were allowed free access to CRF-1 solid food (Charles River, Japan) and tap water free of carcinogens.

### *Surgical procedures*

After fasting for 24 h, animals received intraperitoneal injections of pentobarbital at a dose of 25 mg per kg body weight. Under inhalation anaesthesia with diethyl ether, an upper middle incision was used to perform the following procedures to induce reflux (Figure 1).

*Group A: duodeno-forestomach reflux (*n = *34)* The upper jejunum was transected about 2 cm anal to its origin, and the proximal end was connected to the greater curvature of the forestomach (end-to-side anastomosis). Then the upper duodenum was transected, and the distal end was closed with sutures. The proximal end was anastomosed with the distal end of the transected jejunum. This procedure allowed the duodenal contents to flow back directly into the forestomach and oesophagus through the stoma.

*Group B: duodeno-glandular-forestomach reflux (*n = *33)* The jejunum was transected at the same site as in the group A stoma procedure, but the proximal edge was closed with sutures. The distal cut end was connected to the greater

Correspondence: K. Miwa, Surgery II, School of Medicine, Kanazawa University, Takaramachi 13-1, Kanazawa, 920 Japan.
Received 20 August 1993; and in revised form 15 February 1994.

**Figure 1** Surgical schema. O, oesophagus; F, forestomach; G, glandular stomach; D, duodenum; J, jejunum.

curvature of the forestomach by end-to-side anastomosis. As a result, the duodenal contents flowed back into the forestomach and oesophagus through the glandular stomach.

*Group C: sham operation (n = 33)* The control animals underwent simple laparotomy with blunt manipulation of the stomach and small intestine.

Anastomosis of the gastrointestinal tract was carried out with interrupted sutures of all layers in a line using 7-0 atraumatic silk-braided sutures. Animals surviving 50 weeks post-operatively were killed for examination by exsanguination under anaesthesia.

### Pathology

Immediately after death, the entire stomach was resected along with the oesophagus, duodenum and anastomosed portion of the jejunum. The removed organs were longitudinally incised along the greater curvature and were immediately washed with 10% buffered formalin. With the mucosal surface upward, the margins of the specimen were fixed to a cork plate with pins for macroscopic examination. Specimens were fixed in 10% buffered formalin. Step sections of the oesophagus, forestomach and glandular stomach at 3 mm intervals were prepared in a longitudinal direction so as to include the diameter of the tumours. These sections were embedded in paraffin, cut into 5 μm sections and stained either with haematoxylin-eosin or with haematoxylin-eosin and periodic acid-Schiff (PAS) for microscopic examination.

Histological findings of the squamous epithelium were classified into the following five types:

1. Regenerative thickening: epithelial layer is more than 2-fold thicker than the normal, with acanthosis, elongation of papillae and parakeratosis. The stratified structure of the epithelium is well preserved.
2. Basal cell hyperplasia: thickening of basal layer, constituting more than 15% of the epithelial layer, occasionally resulting in the formation of intramural cysts. The stratified structure is still preserved.
3. Columnar metaplasia: oesophageal mucosa lined by columnar and goblet cells, with absence of squamous cells.
4. Dysplasia: epithelial layer composed of dysplastic squamous cells, with slight to moderate atypia, with pleiomorphic, darkly stained nuclei and an increased number of mitotic figures. There is partial or total absence of stratified structure, but no invasion to the submucosal layer.
5. Carcinomas: tissue composed primarily of anaplastic cells, with marked cellular and structural atypism, with increased mitotic figures, and there is submucosal invasion.

### Hepatobiliary scinti-scanning

Two animals in each group received an i.v. injection of 37 MBq of [$^{99m}$Tc]*N*-pyridoxyl-5-methyltryptophan ($^{99m}$Tc-PMT: Japan Mediphics, Japan) under ether anaesthesia for serial hepatobiliary scanning in the supine position using a gamma camera.

### Statistical analysis

The Fisher exact probability test (Siegel, 1956) was used for statistical analysis of the incidence of abnormal findings, and *P*-values of less than 0.05 were considered significant.

### Results

### General observations

Of 100 operated rats, 61 survived to 50 weeks after surgery. The survival rates for group A (duodeno-forestomach reflux), group B (duodeno-glandular-forestomach reflux) and group C (sham operation) were 32.4% (11/34), 54.5% (18/33) and 97.0% (32/33) respectively. All deaths occurred within 3 months after surgery. The causes of death were emaciation due to reflux oesophagitis in 18 animals, gastric stasis in seven, anastomotic leakage in five, anaesthetic in five and unknown in four.

### Macroscopic findings

The animals of group C showed no abnormalities. In all animals of both groups A and B (reflux groups), the lower oesophagus and forestomach were contracted, with thickening of the wall, and the upper and middle oesophagus were dilated. The lower oesophageal mucosa showed tortuous longitudinal folds and sporadic erosions. The forestomach showed gyrus-like mucosal thickening. Of 11 animals in group A, tumours were found in the lower oesophaguses of two animals and in the forestomachs of three. Of 18 animals of group B, two had oesophageal tumours and seven had tumours in the forestomach. The tumours were whitish nodules and measured between 3 and 5 mm. In addition, tumours of the glandular stomach were found in the prepyloric antrum in eight animals in group B.

### Histological changes

Histology revealed the tumours of the lower oesophagus and forestomach to be either carcinoma or dysplasia. The tumours of the glandular stomach were either adenocarcinoma or adenoma. No lesions appeared among the controls. In animals with reflux, the mucosa of the lower oesophagus and forestomach was 3- to 5-fold thicker than in the control animals. Histological changes in the lower oesophagus, forestomach and glandular stomach are shown in Table I.

*Carcinoma* Carcinomas of the lower oesophagus and forestomach were found in 4/11 (36%) animals in group A and in 3/18 (17%) animals in group B. The differences were significant from group C, which showed no neoplasia ($P<0.01$ and $P<0.05$ respectively). The cancers were squamous cell carcinomas (Figure 2), with the exception of a single PAS-positive mucinous adenocarcinoma (Figure 3) in one animal from group A. The squamous cell carcinomas included both well-differentiated and poorly differentiated types. Carcinoma invasion penetrated up to the submucosal layer and was invariably surrounded by chronic inflammatory changes, including regenerative thickening, basal cell hyperplasia and dysplasia. There was no lymph nodal or remote metastasis. Well-differentiated adenocarcinomas of the glandular stomach were observed in the prepyloric antrum in six of 18 animals (33%) in group B; no neoplasms were found in the glandular stomach in both

**Table I** Incidence of histological changes in the oesophagus, forestomach and glandular stomach of rats

| Group | *A: Duodeno-forestomach reflux* (n = 11) | *B: Duodeno-glandular-forestomach reflux* (n = 18) | *C: Sham operation* (n = 32) |
|---|---|---|---|
| Squamous cell carcinoma | | | |
| Oesophagus | 2 (18) | 0 | 0 |
| Forestomach | 1 (9) | 3 (17)[a] | 0 |
| Adenocarcinoma | | | |
| Oesophagus | 0 | 0 | 0 |
| Forestomach | 1 (9)[b] | 0 | 0 |
| Glandular stomach | 0 | 6 (33)[c] | 0 |
| Dysplasia | | | |
| Oesophagus | 2 (18) | 0 | 0 |
| Forestomach | 6 (55)[d] | 10 (56)[d] | 0 |
| Basal cell hyperplasia | | | |
| Oesophagus | 5 (45)[d] | 6 (33)[d] | 0 |
| Forestomach | 9 (82)[d] | 16 (89)[d] | 0 |
| Regenerative thickening | | | |
| Oesophagus | 8 (73)[d] | 10 (56)[d] | 0 |
| Forestomach | 9 (82)[d] | 17 (94)[d] | 0 |
| Columnar epithelial metaplasia | | | |
| Oesophagus | 2 (18)[b] | 0 | 0 |
| Forestomach | 0 | 0 | 0 |

Data show number of rats, with percentages in parentheses. Every carcinoma of the forestomach and oesophagus was inevitably surrounded by histological changes such as regenerative thickening, basal cell hyperplasis and dysplasia. [a]$P<0.05$ compared with group C. [b]The animal with adenocarcinoma in the forestomach also showed multifocal columnar epithelial metaplasia of the oesophagus. [c]$P<0.01$ and $P<0.05$ compared with groups C and A respectively. [d]$P<0.01$ compared with group C.

**Figure 2** Well-differentiated squamous cell carcinoma in the forestomach of a rat with duodeno-glandular-forestomach reflux. Cancer pearls are present (H&E × 50).

**Figure 3** Mucinous adenocarcinoma in the forestomach of a rat with duodeno-forestomach reflux (H&E × 50).

groups A and C. This difference was significant ($P<0.05$ and $P<0.01$ respectively).

*Associated lesions* Dysplasia (Figure 4) was observed in the lower oesophagus and forestomach of group A animals and in the forestomach of group B animals. The incidence of the dysplasia in the forestomach of animals of both groups A and B was significantly higher than in group C ($P<0.01$ in each group). Dysplasia in the forestomach was frequently found at the anastomotic site.

Basal cell hyperplasia (Figure 5) and regenerative thickening were always observed in the regions surrounding tumours, and erosion was found in animals with reflux. The incidence of basal cell hyperplasia and regenerative thickening in animals in groups A and B was significantly higher than in group C ($P<0.01$ in every case). Basal cell hyperplasia was accompanied by regenerative thickening, which was associated with infiltration of mononuclear cells and eosinophils in the subepithelial layers. These findings were characteristic of chronic reflux oesophagitis. Intramural cysts with hyperplasia were not found in the oesophagus, but were present in the forestomach in ten animals in group A and in six in group B.

In addition, multifocal columnar epithelial metaplasia positive for PAS was found in the base of the epithelial layer of the lower oesophagus in 2 of 11 animals (18%) in group A (Figure 6), one of these two being the animal with adenocarcinoma in the forestomach.

### *Hepatobiliary scinti-scanning*

Two animals from each experimental group underwent scanning. In the two animals from group A, the bile began to flow into the forestomach after 7 and 9 min. Almost the entire volume of bile was present in the forestomach after 40 and 41 min respectively. Reflux into the oesophagus occurred after 60 and 65 min respectively. In the two animals from group B, the material entered the glandular stomach after 20 and 21 min, and reached the forestomach after 23 and 25 min respectively. No oesophageal reflux was observed during the 90 min examination. In the control animals from group C, trace amounts of intragastric reflux occurred, but the substance entered the jejunum after 7 min in both animals.

**Figure 4** Dysplasia in the forestomach of a rat with duodeno-glandular-forestomach reflux (H&E × 100).

**Figure 5** Basal cell hyperplasia in the lower oesophagus of a rat with duodeno-forestomach reflux (H&E × 132).

**Figure 6** Columnar epithelial metaplasia in the lower oesophagus of a rat with duodeno-forestomach reflux. These cells are positive for PAS. The epithelium shows mild dysplasia (H&E × 50).

### Discussion

This study led us to the conclusion that chronic reflux of duodenal contents alone can induce carcinoma in the lower oesophagus and forestomach of rats. The forestomach of the rat is an anatomical extension of the oesophagus and has squamous epithelium that is histologically identical to that of the oesophagus. Rats probably have some type of antireflux barrier between the glandular stomach and the forestomach and between the forestomach and the oesophagus. Nevertheless, duodenal reflux into the stomach and further up into the oesophagus was observed in animals of groups A and B with the scinti-scanning. Some previous induced duodeno-oesophageal reflux studies failed to produce neoplasia in rats without administration of carcinogen (Pera *et al.*, 1989; Seto *et al.*, 1991). The present study has succeeded presumably because of the longer exposure time (50 weeks) compared with previous studies.

Six of the seven neoplasms were squamous cell carcinomas, and one was an adenocarcinoma. The presence of adenocarcinoma confirmed the findings of Attwood *et al.* (1992), who found oesophageal adenocarcinoma in 1 of 20 rats which underwent duodeno-oesophageal anastomosis without carcinogen administration. In addition, the present study demonstrated that squamous cell carcinomas also develop in the lower oesophagus with duodenal content reflux. Every carcinoma was inevitably surrounded by signs of chronic oesophagitis, including regenerative epithelial thickening, basal cell hyperplasia and dysplasia. Therefore, chronic reflux oesophagitis can be considered a cancer-related condition.

Although reflux oesophagitis was induced by a mixture of gastric and duodenal contents in the present investigation, which components of the refluxate, i.e. gastric or duodenal contents, exhibit carcinogenic activity on the squamous epithelium is a crucial question. Duodenal contents have been confirmed as carcinogenic for glandular stomach epithelium in rats by several authors (Langhans *et al.*, 1981; Kondo *et al.*, 1984; Theuring *et al.*, 1985; Mason, 1986; Miwa *et al.*, 1992). In the present study, gastric carcinoma (carcinoma of the prepyloric antrum) only developed in animals whose prepyloric antrum had been directly exposed to duodenal contents (i.e. the duodeno-glandular-forestomach reflux = group B). In addition, hepatobiliary scinti-scanning clearly resolved bile reflux into the forestomach and lower oesophagus, where oesophageal carcinoma developed. Moreover, it has been reported that duodenal contents promote oesophageal carcinogenesis in rats (Pera *et al.*, 1989; Seto *et al.*, 1991; Attwood *et al.*, 1992). In contrast, the gastric contents have never been demonstrated to contain carcinogens that can induce oesophageal carcinogenesis (Attwood *et al.*, 1992). These findings suggest that duodenal contents are more likely than gastric contents to be the cause of reflux-induced oesophageal carcinoma.

Observations in human and animal studies have indicated that columnar-lined epithelial metaplasia in the lower oesophagus is probably caused by chronic reflux of gastric contents (Mossberg, 1966; Bremner *et al.*, 1970, Hamilton & Yardley, 1975). However, the columnar epithelium of Barrett's oesophagus has also been reported to occur after total gastrectomy in which there is no gastric juice to reflux into the oesophagus (Meyer *et al.*, 1979; Sandvik & Havorsen, 1988). Other recent studies (Gillen *et al.*, 1988; Waring *et al.*, 1990) showed that patients with columnar-lined lower oesophagus epithelial metaplasia have higher bile acid levels in the stomach than do either patients with reflux oesophagitis without columnar-lined epithelium or normal individuals. The development of complications (stricture, ulceration and dysplasia) in Barrett's oesophagus are suggested to be related to alkaline gastro-oesophageal reflux (Attwood *et al.*, 1989). These studies suggest that reflux of not only gastric but also duodenal contents which contain bile may play an important role in the development of oesophageal columnar-lined epithelium. In the present study we observed that, with chronic reflux of duodenal contents, columnar-lined epithelial metaplasia arose in the squamous epithelium and that mucinous adenocarcinoma also appeared. Pera *et al.* (1989) and Attwood *et al.* (1992) both reported that, in rats, carcinogens such as 2,6-dimethylnitrosomorphine or methyl-*n*-amylnitrosamine induce oesophageal squamous cell carcinomas under ordinary circumstances but that in the presence of duodeno-oesophageal reflux they may also be associated with adenocarcinomas. Therefore, in the presence of duodenal reflux, the progenitor cells of the oesophageal mucosa seem to have the potential to differentiate not only into normal squamous epithelium and squamous cell carcinoma, but also into columnar epithelial metaplasia and, as a consequence, adenocarcinoma.

Although further investigation is necessary to resolve the exact mechanism by which reflux induces cancer, the following hypothesis is possible. Chemical irritation by refluxed duodeno-gastric juice causes first acute erosive oesophagitis and destruction of normal squamous epithelium. Denudation of the mucosa stimulates progenitor cells in the basal layers, causing acanthosis as a non-specific response. Further stimulation by the refluxed juices on the regenerative epithelium leads to chronic reflux oesophagitis and associated basal cell hyperplasia. Basal cell hyperplasia implies increased replication of mucosa progenitor cells. The increase in the absolute number of proliferative cells in the mucosa may increase susceptibility to carcinogens presumably contained in the luminal refluxed contents. The close association between enlargment of the proliferative compartment and carcinogenesis has already been confirmed in other organs such as skin, large intestine, pancreas and stomach (Iversen *et al.*, 1970; Oehlert, 1973; Lipkin *et al.*, 1983; Williamson & Rainey, 1984; Howatson & Carter, 1985; Miwa *et al.*, 1993).

In conclusion, reflux of duodenal contents is an important contributing factor to oesophageal carcinogenesis. The exact mechanism of this carcinogenesis is unclear and requires further investigation.

### References

ATTWOOD, S.E.A., DEMEESTER, T.R., BREMNER, C.G., BARLOW, A.P. & HINDER, R.A. (1989). Alkaline gastroesophageal reflux: implications in the development of complications in Barrett's columnar-lined lower esophagus. *Surgery*, **106**, 764-770.

ATTWOOD, S.E.A., SMYRK, T.C., DEMEESTER, T.R., MIRVISCH, S.S., STEIN, H.J. & HINDER, R.A. (1992). Duodenoesophageal reflux and the development of esophageal adenocarcinoma in rats. *Surgery*, **111**, 503-510.

BREMNER, C.G., LYNCH, V.P. & ELLIS, Jr. F.H. (1970). Barrett's esophagus: congenital or acquired? An experimental study of esophageal regeneration in the dog. *Surgery*, **68**, 209-216.

CRESPI, M., GRASSI, A., AMIRI, G., MUNOZ, N., ARAMESH, B. & MOJTAHAI, B. (1979). Oesophageal lesions in northern Iran: a premalignant condition. *Lancet*, **ii**, 217-220.

CRESPI, M., GRASSI, A., MUNOZ, N., QUING, W.G. & GUANREI, Y. (1984). Endoscopic features of suspected precancerous lesions in high risk areas of esophageal cancer. *Endoscopy*, **16**, 85-91.

GILLEN, P., KEELING, P., BYRNE, P.J., HEALY, M., O'MOORE, R.R. & HENNESSY, T.P.J. (1988). Implication of duodenogastric reflux in the pathogenesis of Barrett's oesophagus. *Br. J. Surg.*, **75**, 540-543.

GOSWAMI, K.C., KHUROO, M.S., ZARGAR, S.A. & PATHANIA, A.G.S. (1987). Chronic oesophagitis in a population (Kashmir) with high prevalence of oesophageal carcinoma. *Indian J. Cancer*, **24**, 232-241.

GUANREI, Y. & SONGLINIANG, Q. (1987). Endoscopic surveys in high-risk and low-risk populations for esophageal cancer in China with special reference to precursors to esophageal cancer. *Endoscopy*, **19**, 91-95.

HALVORSEN, J.F. & SEMP, B.K.H. (1975). The Barrett syndrome (the columnar-lined lower esophagus): an acquired condition secondary to reflux esophagitis, a case report with discussion of pathogenesis. *Acta Chir. Scand.*, **141**, 683-687.

HAMILTON, S.R. & YARDLEY, J.H. (1975). Regeneration of cardiac type mucosa and acquisition of Barrett mucosa after esophagogastrostomy. *Gastroenterology*, **72**, 669-675.

HOWARTSON, A.G. & CARTER, D.C. (1985). Pancreatic carcinogenesis enhancement by cholecystokinin in the hamster-nitrosamine model. *Br. J. Cancer*, **51**, 107-114.

IVERSEN, V., IVERSEN, O.H., HENNINGS, H. & BJERKNES, R.B. (1970). Diurnal variation in susceptibility of mouse skin to the tumorigneic action of methylcholanthrene. *J. Natl Cancer Inst.*, **45**, 269-276.

KONDO, K., SUZUKI, H. & NAGAYO, T. (1984). The influence of gastrojejunal anastomosis on gastric carcinogenesis in rats. *Gann*, **75**, 362-369.

KUYLENSTIERNA, R. & MUNCK-WIKLAND, E. (1985). Esophagitis and cancer of the esophagus. *Cancer*, **56**, 837-839.

LANGHANS, P., HEGER, R.A., HOHENSTEIN, J., SCHLAKE, W. & BÜNTE, H. (1981). Operation-sequelae carcinoma of the stomach. Experimental studies of surgical techniques with or without resection. *World J. Surg.*, **5**, 595-605.

LIPKIN, M., BLATTNER, W.E., FRAUMENI, J.F., LYNCH, H.T., DESCHNER, E. & WINAWER, S. (1983). Tritiated thymidine labeling distribution as a marker for hereditary predisposition to colon cancer. *Cancer Res.*, **43**, 1899-1904.

McDONALD, G.B., BRAND, D.L. & THORNING, D.R. (1977). Multiple adenomatous neoplasms arising in columnar-lined (Barrett's) esophagus. *Gastroenterology*, **72**, 1317-1321.

MAETA, M., KOGA, S., SHIMIZU, T. & MATSUI, K. (1990). Possible association between gastrectomy and subsequent development of esophageal cancer. *J. Surg. Oncol.*, **44**, 20-24.

MASON, R.C. (1986). Duodenogastric reflux in rat gastric carcinoma. *Br. J. Surg.*, **73**, 801-803.

MEYER, W., VOLLMAR, F. & BARR, W. (1979). Barrett oesophagus following total gastrectomy. *Endoscopy*, **11**, 121-126.

MIROS, M., KERLIN, P. & WALKER, N. (1991). Only patients with dysplasia progress to adenocarcinoma in Barrett's esophagus. *Gut*, **32**, 1441-1446.

MIWA, K., FUJIMURA, T., HASEGAWA, H., KOSAKA, T., MIYATA, R., MIYAZAKI, I. & HATTORI, T. (1992). Is bile or are pancreaticoduodenal secretions related to gastric carcinogenesis in rats with reflux through the pylorus? *J. Cancer Res. Clin. Oncol.*, **118**, 570-574.

MIWA, K., KAMATA, T., MIYAZAKI, I. & HATTORI, T. (1993). Kinetic changes and experimental carcinogenesis after Billroth I and II gastrectomy. *Br. J. Surg.*, **80**, 893-896.

MOSSBERG, S.M. (1966). The columnar-lined esophagus (Barrett syndrome): an acquired condition? *Gastroenterology*, **50**, 671-676.

MUNOZ, N., CRESPI, M., GRASSI, A., QING, W.G., QUIONG, S. & CAI, L.Z. (1982). Precursor lesions of oesophageal cancer in high-risk populations in Iran and China. *Lancet*, **i**, 876-879.

NAEF, A.P., SAVARY, M. & OZELLO, L. (1975). Columnar lined lower esophagus: an acquired lesion with malignant predisposition. *J. Thorac. Cardiovasc. Surg.*, **70**, 826-835.

OEHLERT, W. (1973). Cellular proliferation in carcinogenesis. *Cell Tissue Kinet.*, **6**, 325-335.

OETTLE, G.J., PATERSON, A.C., LEIMAN, G. & SEGAL, I. (1986). Esophagitis in a population at risk for esophageal carcinoma. *Cancer*, **57**, 2222-2229.

PERA, M., CARDESA, A., BOMBI, J.A., ERNST, H., PERA, C. & MOHR, U. (1989). Influence of esophagojejunostomy on the induction of adenocarcinoma of the distal esophagus in Sprague-Dawley rats by subcutaneous injection of 2,6-dimenthylnitroso-morphine. *Cancer Res.*, **46**, 6803-6808.

ROSSI, M., ARCONA, E., FINCO, C. & PERACCHIA, A. (1984). Esophageal cancer and previous partial gastrectomy. *Int. Surg.*, **69**, 369-370.

SANDVIK, A.K. & HAVORSEN, T.B. (1988). Barrett's esophagus after total gastrectomy: a contribution of its pathogenesis. *J. Clin. Gastroenterol.*, **10**, 587-588.

SEABROOK, M., HOLT, S. & GILRANE, T. (1992). Barrett's esophagus: observations on diagnosis and management. *South. Med. J.*, **85**, 280-288.

SETO, Y., KOBORI, O., SHIMIZU, E. & MORIOKA, Y. (1991). The role of alkaline reflux in esophageal carcinogenesis induced by N-amyl-N-methylnitrosamine in rats. *Int. J. Cancer*, **49**, 758-763.

SHEARMAN, D.J.C., FINLAYSON, N.D.C., ARNOTT, S.J. & PEARSON, J.G. (1970). Carcinoma of the esophagus after gastric surgery. *Lancet*, **i**, 581-582.

SIEGEL, S. (1956). *Non-Parametric Statistics for the Behavioral Sciences*. McGraw-Hill: New York.

THEURING, F., DITTRICH, S. & WOLTER, F.H. (1985). On the varying degrees of cancerogenicity of modified gastroentero-anastomoses. *Exp. Pathol.*, **27**, 179-184.

WARING, J.P., LEGRAND, J., CHINICHIAN, A. & SANOWSKI, R.A. (1990). Duodenogastric reflux in patients with Barrett's esophagus. *Dig. Dis. Sci.*, **35**, 759-762.

WILLIAMSON, R.C.N. & RAINEY, J.B. (1984). The relationship between intestinal hyperplasia and carcinogenesis. *Scand. J. Gastroenterol.*, **19** (Suppl. 104), 57-76.

WITT, T.R., BAINS, M.S., ZAMANB, M.B. & MARTINI, N. (1983). Adenocarcinoma in Barrett's esophagus. *J. Thorac. Cardiovasc. Surg.*, **85**, 337-345.